# Resection of bilateral massive Achilles tendon xanthomata with reconstruction using vascularized iliotibial tract: A case report and literature review

**DOI:** 10.1097/MD.0000000000036247

**Published:** 2023-12-08

**Authors:** Jian Qi, Long Fang, Wei Hao, Lin Zou

**Affiliations:** a Department of Orthopedics, The 960th Hospital of PLA, Jinan, China; b Department of Orthopedics and Traumatology, Shandong Provincial Third Hospital, Shandong University, Jinan, China.

**Keywords:** Achilles tendon, iliotibial tract, reconstruction, vascularized, xanthomata

## Abstract

**Rationale:**

Cerebrotendinous xanthomatosis is a rare autosomal recessive metabolic disease. Surgical treatment is only indicated when the xanthoma becomes large, painful, and irritable with shoe wear. Reconstruction of the large defect following resection challenging, especially with resection of the entire Achilles tendon.

**Patient concerns:**

We report a case of bilateral Achilles tendon defects of 16 cm following resection of bilateral Achilles tendon xanthomata, with reconstruction using vascularized iliotibial tract. The patient had a good functional outcome with well-preserved strength and cosmesis.

**Outcomes:**

Reconstruction of a total Achilles tendon defect using Vascularized iliotibial tract is safe and effective.

## 1. Introduction

Cerebrotendinous xanthomatosis is a rare autosomal recessive metabolic disease. It caused by mutations in the mitochondrial enzyme, sterol 27-hydroxylase (CYP27A1) gene,^[1]^and classically characterized by bilateral Achilles tendon xanthoma; bilateral cataract formation; and progressive neurological dysfunction with mainly pyramidal tract signs, cerebellar ataxia, and cognitive impairment.^[2]^ Diagnosis can be confirmed by high plasma cholestanol levels and elevated bile alcohols in urine.^[3]^

Traditionally, the treatment of Achilles tendon xanthomata involved 1 of 2 methods: non-operative with bile-acid therapy which is effective, affordable and safe; alternatively, surgery can be undertaken for tumor resection and Achilles tendon reconstruction. Non-operative therapy has a significant disadvantage as it does not address the deformity of tendon region which often results in shoe wear difficulties. Surgery for tumor resection and Achilles tendon reconstruction is an alternative to address these issues but entire loss of Achilles tendon presents a reconstructive challenge. Surgical treatment is indicated when the xanthoma becomes large, painful, and irritable from friction against the shoe.^[4–6]^

There are several techniques reported to reconstruct Achilles tendon after tumor resection, including FHL transfer,^[7,8]^ FDL transfer,^[9]^ peroneus brevis tendon transfer,^[7,10]^ autogenous peroneus longus tendon graft,^[11]^ V-Ygastrocnemius tendon flap,^[12]^ plantar tendon transfer,^[13]^ fascia lata flap,^[14]^ synthetic material graft^[15–20]^and Cadaver tendon/bone-tendon graft.^[21]^ Each of the numerous methods currently used have certain advantages and disadvantages. In our knowledge, there are only 4 articles describing use of the entire iliotibial tract to replace the Achilles tendon,^[22–25]^ and only one has been reported in the English language literature.^[25]^

This article reports the use of the iliotibial tract to reconstruct an entire defect of Achilles tendon. We review the diagnosis, clinical manifestations, physical examination, radiological and laboratory findings. The surgical technique involved bilateral Achilles tendon xanthomatas resection with total loss of Achilles tendons. The segmental defects were reconstructed using bilateral iliotibial tract as graft.

## 2. Case report

A 32-year-old woman presented to our hospital on April 2005 for tumors in both Achilles regions that had been slowly growing for 8 years. She noted painful swelling of the Achilles tendons and deformity. There was no history of trauma nor a family history for this disorder. She reported no neurological symptoms and no other inherited disorders.

**Figure 1.**
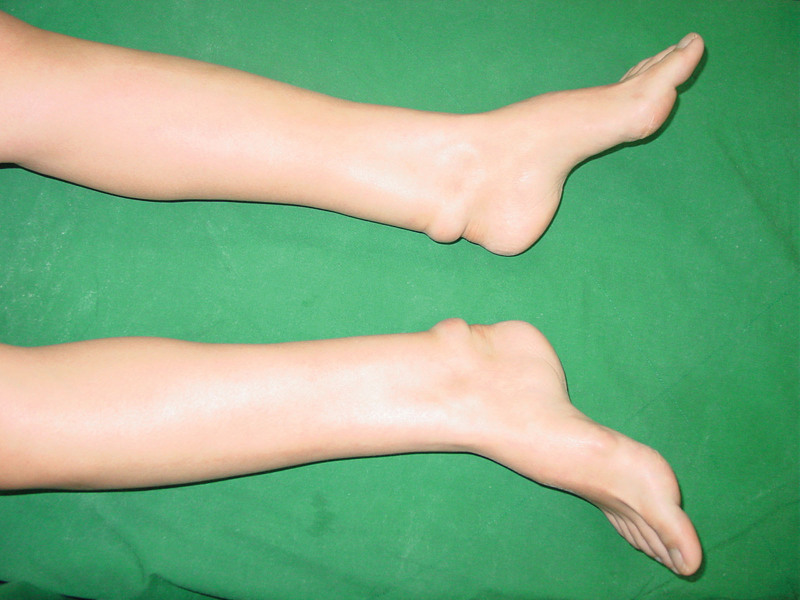
Clinical photograph demonstrating bilateral symmetrical fusiform swelling of the Achilles tendons.

**Figure 2.**
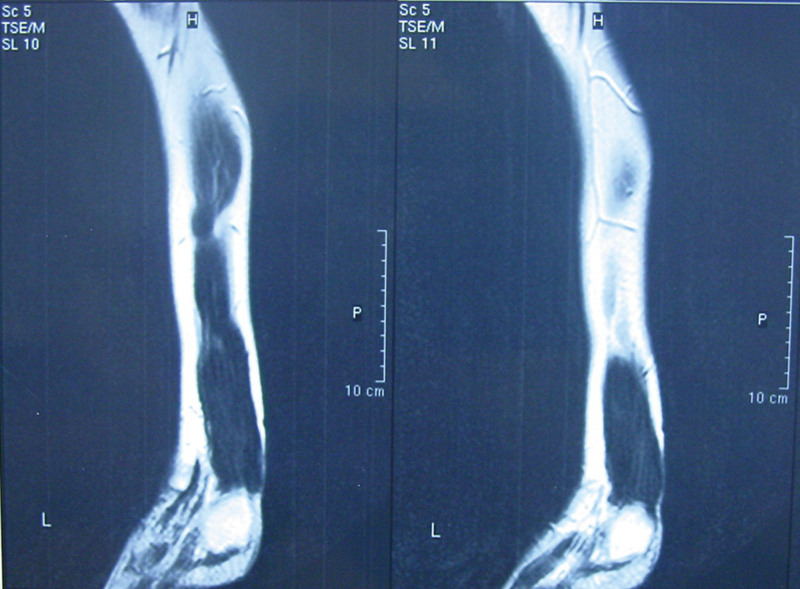
MRI showing significant enlargement of both Achilles tendons with fusiform swelling. MRI = magnetic resonance imaging.

Physical examination showed a moderate obesity of the patient (height, 161cm; weight, 60.1 kg). There were firm, non-tender and diffuse tumors in her both Achilles tendons. The deformity was distributed over the distal 16cm segment of each tendon (Fig. 1). She had a normal gait and the feet had no deformity, although both ankles had dorsiflexion and plantarflexion limitation to 10° and 20°. There were no other nodules or skin lesions observed in any other limb. Magnetic resonance imaging (Fig. 2) showed significant enlargement of both Achilles tendons with fusiform swelling. The serum cholestanol level was 2.4 mg/dL, which is twelve times the normal mean value of 0.2 ± 0.2 mg/dL, which confirmed the diagnosis of cerebrotendinous xanthomatosis.

## 3. Surgical procedure

The patient underwent 2 staged surgeries. First, the left Achilles tendon xanthomatas was resected and reconstructed using right iliotibial tract graft. After 15 days, the right Achilles tendon xanthomatas was resected and reconstructed using left iliotibial tract graft.

**Figure 3.**
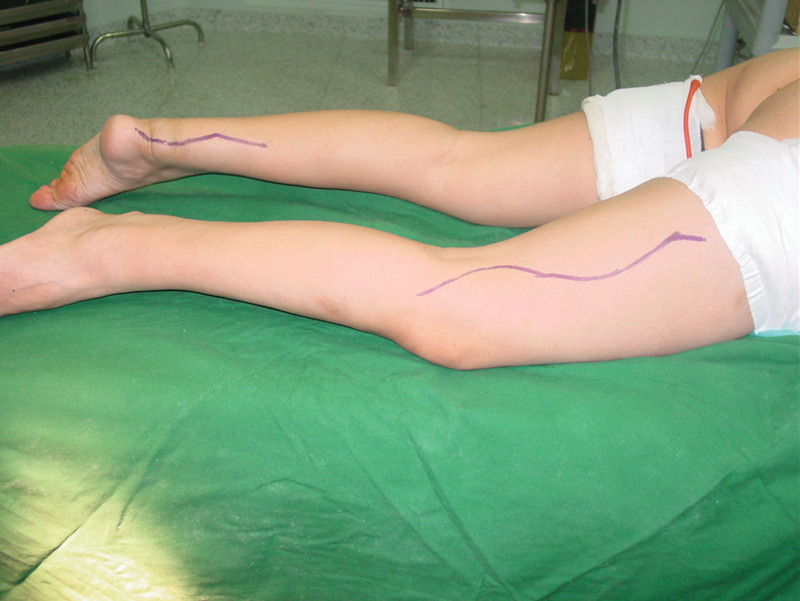
Photograph showing incision designed.

**Figure 4.**
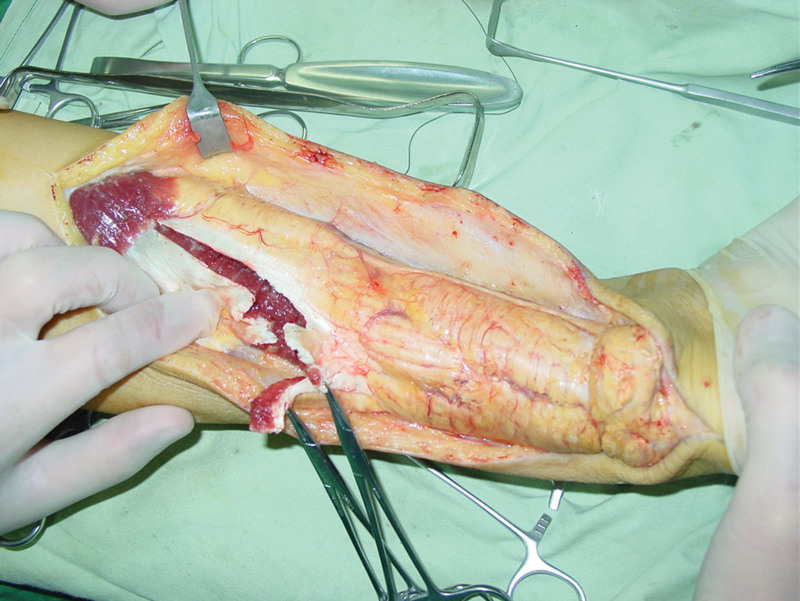
Photograph showing from the musculotendinous junction to the insertion almost changed to a long yellow Achilles tendon xanthomata.

**Figure 5.**
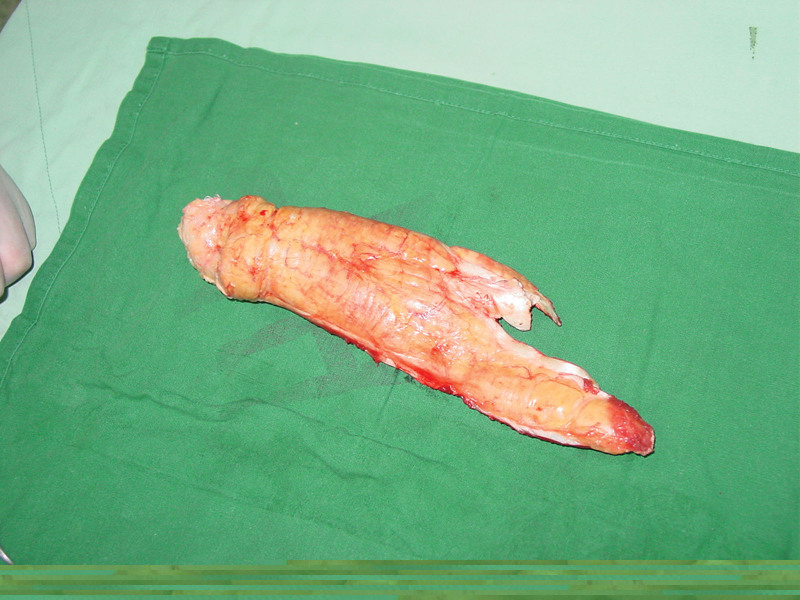
The Achilles tendon xanthomatas was resected.

The procedures were performed with the patient under epidural anesthesia. The patient was placed in a prone position with at high tourniquet inflated to 300 mm Hg. The skin and Achilles tendon envelope were incised through posterior midline approach (Fig. 3). The Achilles tendon from the musculotendinous junction to the insertion was noted to be infiltrated with a yellow-colored Achilles tendon xanthomatas (Figs. 4 and 5). The tumor was resected in total and a significant segmental Achilles tendon defect remained, approximately 16 cm in length.

**Figure 6.**
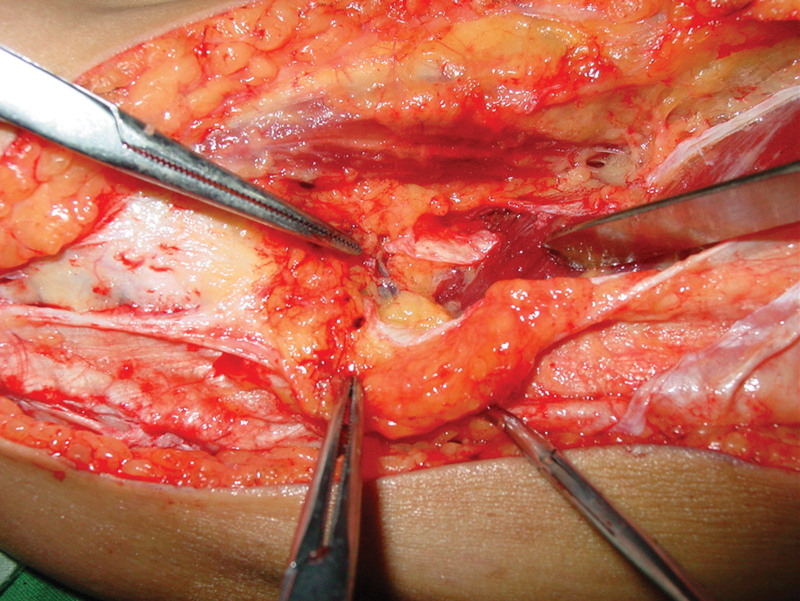
Clinical photograph showing the location of the lateral superior genicular vessel.

The iliotibial tract was harvested through longitudinal arc-shaped incision along the posterior lateral aspect of the thigh above knee, approximately 16 cm in length. Subcutaneous tissue and deep fascia were dissected from the iliotibial tract, entered into spatium intermusculare through the lateral border of caput breve musculi bicipitis femoris. The knee was flexed to mobilize the caput breve musculi bicipitis femoris and expose the lateral superior genicular vessel at about 5cm above caput fibulae superior border (Fig. 6). The main vascular truck was dissociated carefully and other branches were ligated at this level. Approximately 2 cm extra iliotibial tract was taken at both sides, to provide adequate fixation to the musculotendinous junction of the Achilles tendon and calcaneal bone, for a total of 20 cm in length. Since the strength of the repair was determined by the width of the iliotibial tract to provide a strong tendon repair, a strip 4 cm in width was harvested. After that the dissection was continued at the periosteum surface of lateral condyle of femur to release the lateral superior genicular vessel until ascending branch and/or descending branch entering into the iliotibial tract flap were identified.^[22]^ The pedicle was divided and the Vascularized iliotibial tract flap was prepared to graft.

**Figure 7.**
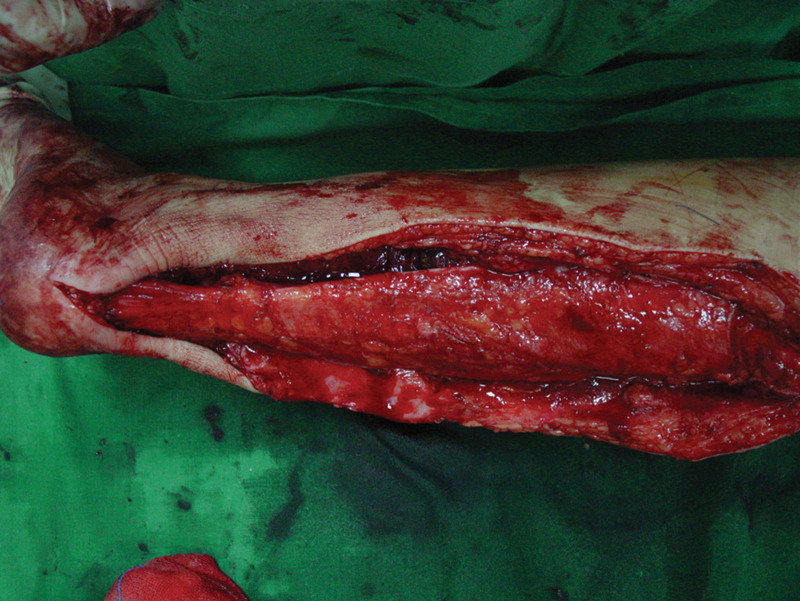
Clinical photograph showing the iliotibial tract was rolled into a tendon-like structure and attached to the musculotendinous junction, the distal was fixed into calcaneal tuberosity.

The flap was transferred to the defect site. The iliotibial tract was rolled into a tendon-like structure along its longitudinal axis. The proximal portion of the iliotibial tract was attached to the musculotendinous junction of the Achilles tendon with 2–0 Prolene sutures, the distal end was fixed into calcaneal tuberosity using an absorbable interference screw (Fig. 7). This was done with the iliotibial tract graft under some tension and the ankle in neutral position. Next, microvascular anastomosis was performed between the flap pedicle and the posterior tibial vessel, preferably with the posterior tibial vessel because of its proximity to the injury site.^[26]^ After confirmation of moderate Achilles tendon tension, we cleaned and sutured the wound of the donor site and Achilles site. The same reconstructive measures were applied to the Achilles tendon on the contralateral side after 15 days.

Postoperatively, the affected leg is immobilized in 10 degrees plantarflexion with a below-knee full cast for 6 weeks. A removable boot was applied for a further 6 weeks. At the first week, the patient was managed with antibiotic (3 days), antispasmodic and antithrombotic agent. In the initial 2 weeks, color Doppler ultrasound was used to confirm anastomosis patency. Range of motion exercises were commenced at 8 weeks. Passive motion was started followed by the active range of motion including heel-raises and walking. Athletic activity was restricted for 6 months after surgery.

**Figure 8.**
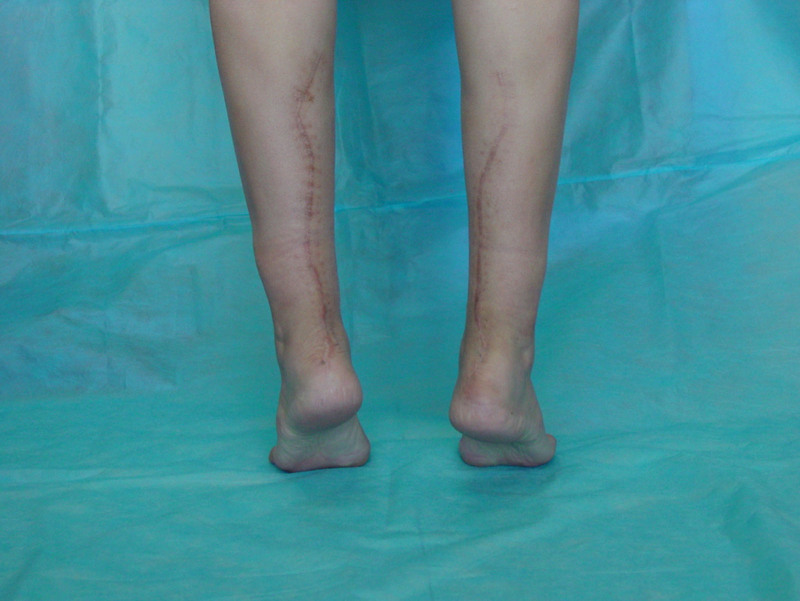
Photograph showing patient can stand on tip toes without support.

**Figure 9.**
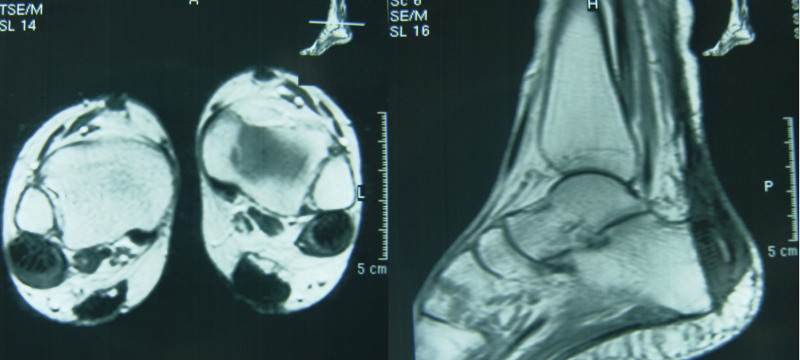
Magnetic resonance imaging (MRI) scan of the reconstructed Achilles tendon postoperative 2.5 yr. Both axial and saggital magnetic resonance image of the reconstructed Achilles tendonshowing a tendon-like structure.

The patient had an uneventful post-operative recovery, with no neurological or skin complications. There was no scar tenderness, wound hematoma or knee movement restriction at the donor site. After surgery our patient showed good functional results. Her range of ankle motion is from 15°of dorsiflexion to 45°of plantar flexion bilaterally. She could walk comfortably and stand on tiptoes without support (Fig. 8); normal shoe wear could be worn. Three years after the surgery, the patient has undergone Magnetic resonance imaging scan of the reconstructed Achilles tendon. Both axial and sagittal magnetic resonance images of the reconstructed Achilles tendon showed a tendon-like structure (Fig. 9). At final follow up her AOFAS scores 100/100. Currently, the patient is 9 years from her first surgery, with no pain, no limitation of activities, excellent strength and return to her original level of sports activity.

## 4. Discussion

Achilles tendon xanthomata is a rare disorder and the initial presentation may be symmetric, painful enlargement and deformity of the Achilles tendons.^[2]^ Early diagnosis is the key to treatment because medical therapy is effective in halting progression of, although not reversing, the devastating neurological lesions of this condition.^[2]^ Without early diagnosis of the disease, Achilles tendon xanthomata can enlarge slowly and result in painful, deformity which requires surgery to resect the tumor. Segmental loss of the Achilles tendon presents a reconstructive challenge due to the lack of suitable tissue locally capable of transmitting forces of up to 12.5 times body weight.^[26]^ Numerous techniques have been proposed for repair of Achilles tendon defects,^[7–21]^ with each carrying its own relative benefits and disadvantages.

Inadequate strength is the main disadvantage of FHL transfer^[7,8]^ or FDL transfer,^[9]^ peroneus brevis tendon transfer,^[7,10]^ autogenous peroneus longus tendon graft,^[11]^ V-Ygastrocnemius tendon flap,^[12]^ plantar tendon transfer,^[13]^ fascia lata flap^[14]^ and Cadaver tendon/bone-tendon graft,^[21]^ and posterior tibial tendon transfer or autograft.^[27]^ Furthermore, harvesting of the FHL tendon does often result in significant weakness of hallux plantar flexion; use of the flexor digitorum brevis muscle may limit plantar flexion of the toes and create an unwanted mass prone to increased pressure as the muscle is folded back on itself. It is similar as using other tendon transfer. Free fascia lata graft harvested from the thigh was reported by Lee and Weiss.^[5]^ Tomita et al^[28]^ used the same technique and reported on the macroscopic and histological appearance at re-operation after thirty months. Large fascia lata grafts may cause donor site more morbidity in the thigh. Allograft can substitute for segmental loss, it can substitute a long segment loss and gives good functional outcome,^[30]^ but may have increased risk of infection. Use of cadaveric allograft, there exists the potential for viral disease transmission. Moreover, the limited availability of cadaveric grafts in most countries makes this technique impractical. As with any synthetic material graft,^[15–20]^ the strength maybe enough but this foreign material does pose a risk for later infection. Their durability against fatigue failure has also not been proven.

These methods may be unsuitable to reconstruct the total Achilles tendon defects such as those seen in the current patient. So how to avoid the disadvantages and choice a better technique to reconstruct Achilles tendon defect after Achilles tendon xanthomata resection. The author think a good technique of Achilles tendon reconstruction should include those features: it is a single-stage and anatomic reconstruction, there is no or less morbidity and/or deformity of donor site, it has higher resistance to infection, it has no affect of wearing shoes, it has fewer adhesion and good shape of heel region, the reconstructed tendon has enough strength and strong, and more important it has a better function.

Based on the experience with the fascia lata flap, we focused on iliotibial tract. Zhu et al^[29]^ reported biomechanical assessment of several autografts which used to reconstruct Achilles tendon and the results showed that the mechanical properties of the iliotibial tract and Achilles tendon are similar. Specifically, a 2.2 cm wide iliotibial tract can sustain 55 kg tensile stress, peak extensibility of iliotibial tract is 11.5%, breaking strength is 3.9 kg/mm^2^. It is a ideal material for Achilles tendon reconstruction. In this case, we harvested 4cm wide iliotibial tract and tabularized it with a suture to grossly approximate a structure similar to the Achilles tendon.

Iorio^[30]^ reported outcomes in the vascularized and avascular subgroups of tendon graft and found no significant difference in the areas of overall motion and plantar motion. Nevertheless, vascularized tendon substitutes have the advantages of higher resistance to infection, faster healing, fewer adhesions, and better gliding capability. As a result, we elected to use a vascularized iliotibial tract to reconstruct the defect after total Achilles tendon resection for xanthomata.

Reconstruction of a total Achilles tendon defect using Vascularized iliotibial tract was safe and effective. We believe this technique of reconstruction after resection of Achilles tendon xanthomata provides multiple advantages over previous techniques described. These advantages include single-stage and anatomic reconstruction, fast healing, acceptable donor site morbidity or deformity, higher resistance to infection, no detrimental affected for shoe wear, fewer adhesion and good cosmetic appearance, and good retained strength and functional outcome.

## Author contributions

**Data curation:** Jian Qi, Wei Hao, Lin Zou.

**Formal analysis:** Jian Qi, Wei Hao, Lin Zou.

**Investigation:** Long Fang.

**Methodology:** Long Fang.

**Software:** Long Fang.
